# What Is the Best Preoperative Imaging for Endometrial Cancer?

**DOI:** 10.1007/s11912-016-0506-0

**Published:** 2016-02-27

**Authors:** Ingfrid S. Haldorsen, Helga B. Salvesen

**Affiliations:** Department of Radiology, Haukeland University Hospital, Jonas Liesvei 65, Postbox 7800, 5021 Bergen, Norway; Section for Radiology, Department of Clinical Medicine, University of Bergen, 5020 Bergen, Norway; Department of Obstetrics and Gynecology, Haukeland University Hospital, 5020 Bergen, Norway; Department of Clinical Science, University of Bergen, 5020 Bergen, Norway

**Keywords:** Endometrial cancer, Preoperative imaging, Staging, Vaginal ultrasound, Computed tomography, Magnetic resonance imaging, Diffusion weighted imaging, Positron emission tomography, Imaging biomarkers

## Abstract

Although endometrial cancer is surgicopathologically staged, preoperative imaging is recommended for diagnostic work-up to tailor surgery and adjuvant treatment. For preoperative staging, imaging by transvaginal ultrasound (TVU) and/or magnetic resonance imaging (MRI) is valuable to assess local tumor extent, and positron emission tomography-CT (PET-CT) and/or computed tomography (CT) to assess lymph node metastases and distant spread. Preoperative imaging may identify deep myometrial invasion, cervical stromal involvement, pelvic and/or paraaortic lymph node metastases, and distant spread, however, with reported limitations in accuracies and reproducibility. Novel structural and functional imaging techniques offer visualization of microstructural and functional tumor characteristics, reportedly linked to clinical phenotype, thus with a potential for improving risk stratification. In this review, we summarize the reported staging performances of conventional and novel preoperative imaging methods and provide an overview of promising novel imaging methods relevant for endometrial cancer care.

## Introduction

Endometrial cancer is the most common gynecologic malignancy in high-income countries, and the incidence is increasing [1]. Most patients are diagnosed at an early stage with tumors still confined to the uterine corpus in around 75 %. However, after primary surgery, around 15–20 % of these tumors recur in the vagina/pelvis (∼ one third of recurrences) or at distant sites (∼ two thirds of recurrences) [2]. The overall 5-year survival of endometrial cancer for all stages is around 80 % [3]; however, in the metastatic setting, the prognosis is dismal with reported median survival of only 7–12 months [4].

Adjuvant treatment and follow-up after primary surgery for endometrial cancer has since 1988 been guided according to the surgical International Federation of Gynecology and Obstetrics (FIGO) staging systems, which was last revised in 2009 [5]. In addition to risk classification based on investigation of preoperative uterine biopsies, conventional imaging methods, i.e., transvaginal ultrasound (TVU), magnetic resonance imaging (MRI), computed tomography (CT), and positron emission tomography-CT (PET-CT) have, however, long been employed at many centers in order to improve the optimization of risk classification to tailor primary surgical procedure and systemic therapeutic strategy [6]. Although these imaging methods may provide information about likely tumor stage based on conventional imaging findings (e.g., signs of deep myometrial invasion, cervical stromal invasion, and pelvic and/or paraaortic lymph node metastases), the reported accuracies for preoperative staging of endometrial cancer by conventional imaging have not yet been good enough to be accepted to replace surgical staging including lymphadenectomy, in particular for high-risk histology, at many centers.

Novel functional imaging methods within US, MRI and PET-CT, have long gained increasing interest, representing promising additional imaging tools in the characterization of various cancers, including endometrial cancers [7•, 8, 9•, 10–14, 15•, 16]. These advanced imaging methods may enable visualization and quantification of functional and microstructural tumor characteristics that may be closely linked to clinical phenotype, tumor stage, prognostic histomorphological tumor markers, and eventually outcome [9•, 10, 13, 14, 17]. Thus, both conventional and functional imaging may potentially provide preoperative imaging biomarkers in endometrial cancer relevant for treatment and prognosis that could be translated into the clinic to improved risk stratified for individualizing patient treatment. This has the potential to increase clinical benefit through reducing costs and side effects from unnecessary overtreatment in low-risk patients in combination with maintaining the optimal and more comprehensive therapeutic strategy for high-risk patients.

This review provides an overview of current conventional and novel imaging methods for preoperative staging of endometrial cancer and their corresponding reported staging performances. The promising role of novel functional imaging methods to yield potential new imaging biomarkers for improved preoperative risk stratification in endometrial cancer is also discussed.

## Treatment and Staging of Endometrial Cancer

Primary surgical treatment of endometrial cancer is clinically guided by a range of approaches to predict surgical FIGO stage by estimating risk for lymph node metastases and distant spread from endometrial biopsies and preoperative imaging (Table 1). For putative FIGO stage I (tumor confined to the uterine corpus) in low-risk endometrial cancer (endometrioid adenocarcinoma grades 1 and 2 with myometrial infiltration <50 %), with low risk for lymph node spread (Table 1), the surgical procedure is normally limited to simple hysterectomy and bilateral salpingo-oophorectomy (BSO), whereas in intermediate- and high-risk endometrial cancer (endometrioid adenocarcinomas, grades 1 and 2 with myometrial infiltration ≥50 %; endometrioid adenocarcinomas, grade 3; and non-endometrioid adenocarcinomas), pelvic and paraaortic lymphadenectomy/lymph node sampling may be included and sometimes also omentectomy [2]. For endometrial cancers invading the cervical stroma (FIGO stage II), extended hysterectomy, BSO, and pelvic lymphadenectomy are recommended. For putative FIGO stage III (local or regional tumor spread) and IV (distant metastases and/or invasion of the bladder/bowel), surgical treatment is typically individualized consisting of surgical tumor resection including debulking of lymph nodes and metastatic lesions sometimes after neoadjuvant therapy [2].

**Table 1:** Reported impact of histopathologic diagnosis and selected additional biomarkers in endometrial cancer for preoperative prediction of extra uterine disease (EUD) and postoperative prediction of survival. The increase (%) of patients with EUD at diagnosis, and decrease in 5-year survival (%) based on pre- and postoperative assessments of biomarkers are listed

Biomarker	EUD (%)	Decrease in 5-year survival (%)	
		Preoperative biopsy	Hysterectomy specimens
Non-endometrioid histology [1, 18–22]	50	23	40
Grade 3 [19–22]	11–18	17	28
Vascular invasion [21, 22]	20	NR	30
Loss of ER/PR expression [18, 23, 24]	24–27	30	20–30
P53 overexpression [25, 26]	23–49	21–38	30
Aneuploidy [19, 27–30]	22–25	12–22	19

References [18, 19] and [28] are prospective studies

*EUD* extra uterine disease including patients with lymph node metastases, *NR* not reported, *ER* estrogen receptor, *PR* progesterone receptor, *P53* tumor protein p53

Surgical FIGO stage is the single strongest prognostic factor in endometrial cancer with a significant decrease in disease-specific survival in patients with the higher stages. Reported figures on 5-year survival for the different FIGO stages are 90–96 % in stage IA (tumor confined to the uterus with tumor infiltrating <50 % of the myometrial wall), 78–87 % in stage 1B (tumor confined to the uterus with tumor infiltrating ≥50 % of the myometrial wall), 48–56 % in stage II (tumor invading cervical stroma), 48–60 % in stage III (local or regional tumor spread), and ∼20 % in stage IV (distant metastases and/or invasion of the bladder/bowel) [31, 32].

Final risk estimation based on postoperative assessment of hysterectomy specimen for histological subtype and grade, and presence of deep myometrial invasion, cervical stroma involvement and/or metastatic spread, guides the selection of presumed high-risk patients subjected to adjuvant chemo- and/or radiotherapy [2].

## Conventional Diagnostic Imaging for Preoperative Staging of Endometrial Cancer

Preoperative imaging is an essential part of the diagnostic work-up in endometrial cancer and is pivotal to define a presumed FIGO stage (based on imaging findings) guiding the primary surgical treatment in addition to other biomarkers (Table 1). The diagnostic performances of preoperative imaging methods for the identification of deep myometrial invasion, cervical stroma invasion, and extrauterine disease including lymph node metastases preoperatively (Table 2) are critical if they are to safely guide a tailored surgical approach in order to avoid unnecessary invasive procedures in low-risk patients.

**Table 2:** Reported staging performance of conventional and novel imaging methods in endometrial cancer

Imaging method	Deep myometrial invasion					Cervical stroma invasion					Metastatic lymph nodes				
	Sens. (%)	Spec. (%)	Acc. (%)	PPV (%)	NPV (%)	Sens. (%)	Spec. (%)	Acc. (%)	PPV (%)	NPV (%)	Sens. (%)	Spec. (%)	Acc. (%)	PPV (%)	NPV (%)
TVU [33, 34, 35•, 36, 37]	71–85	72–90	72–84	51–79	83–88	29–93	92–94	78–92	48–72	82–98	NR	NR	NR	NR	NR
3D TVU [38]	93	83	85	68	97	88	NR	NR	NR	NR	NR	NR	NR	NR	NR
CE CT [6, 39]^a^	40–100	67–100	NR	NR	NR	47–71	100	NR	NR	NR	29	100	79	NR	NR
CE MRI [37, 40, 41]	33–100	44–100	58–100	51–60	68–89	33–69	82–96	46–89	36–63	85–94	17–80	88–100	83–93	38–100	88–97
DW MRI [8, 40]	63–100	56–100	74–98	59–89	69–76	44–56	92–96	86–88	56–64	90–92	38–86	92–97	87–94	46–63	92
USPIO MRI [42]	NR	NR	NR	NR	NR	NR	NR	NR	NR	NR	91–100	87–94	88–95	71–82	82–88
FDG PET-CT [7•, 37, 43]	93	49	61	41	95	25–43	74–94	66–83	49–69	85–86	63–85	91–96	89–93	59–76	96–98

*Acc*. accuracy, *CE* contrast enhanced, *CT* computed tomography, *DW* diffusion weighted, *FDG* fluorodeoxyglucose, *MRI* magnetic resonance imaging, *NPV* negative predictive value, *NR* not reported, *PET* positron emission tomography, *PPV* positive predictive value, *Sens*. sensitivity, *Spec*. specificity, *TVU* transvaginal ultrasound, *USPIO* ultrasmall particles of iron oxide

^a^Data for myometrial invasion and cervical stroma invasion apply to the 1988 FIGO (International Federation of Gynecology and Obstetrics) staging system

### Transvaginal Ultrasound

**Fig. 1:**
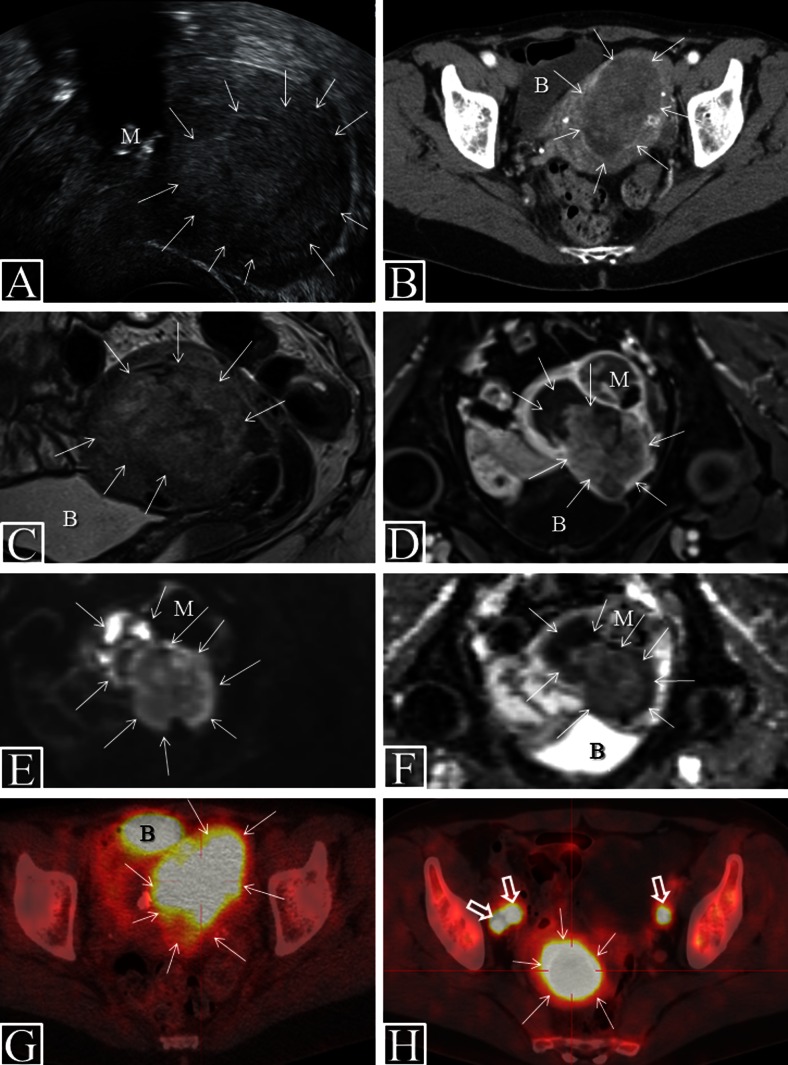
Characteristic preoperative imaging findings in endometrial cancer. VUS (**a**) in patient with FIGO stage 1B (endometrioid, grade 2) depicting a large uterine tumor (*arrows*) with mixed echogenicity and signs of deep myometrial invasion. CE CT (**b**), sagittal T2 weighted MRI (**c**), axial CE T1-weighted MRI (**d**), axial DWI (*b* = 1000 s/mm^2^) (**e**) with ADC map (**f**), and FDG PET-CT (**g**) in a patient with FIGO stage 1B (endometrioid, grade 3). The large uterine tumor (*arrows*), invading >50 % of the myometrial wall, is hypodense relative to the surrounding myometrium at CT (**b**), hyperintense at T2-weighted MRI (**c**), and hypointense at CE T1-weighted MRI. DWI shows tumor hyperintensity (**e**) with corresponding hypointensity on the ADC map (**f**; mean ADC value of 0.54 × 10^−3^ mm^2^/s), indicating restricted diffusion within the tumor. The same lesion is FDG avid at FDG PET-CT (**g**; SUVmax of 10.4). FDG PET-CT in patient with FIGO stage 3C2 (endometrioid, grade 3) (**h**) depicts large FDG avid tumor (SUVmax of 25.0) and three pelvic lymph node metastases (**h**, *open arrows*; SUVmax of 11.3). The bladder (*b*) normally appears FDG avid due to FDG secretion in the urine (**g**). Note the concomitant calcified myoma (*m*) seen at VUS (**a**) and the adjoining myoma (*m*) depicted at MRI (**d**–**f**) with no restricted diffusion; the myoma is thus easy to differentiate from the uterine tumor. *ADC* apparent diffusion coefficient, *B* bladder, *CE* contrast enhanced, *CT* computed tomography, *DWI* diffusion weighted imaging, *FDG* fluorodeoxyglucose, *M* myoma, *MRI* magnetic resonance imaging, *PET* positron emission tomography, *SUV* standard uptake value, *VUS* vaginal ultrasound

TVU is typically performed by the treating gynecologist with the advantage of being readily available and with low costs. Valid ultrasound diagnostics is, however, inherently dependent on a skilled examiner being able to obtain representative images depicting the pathology of interest, and TVU may thus be especially prone to interobserver variation. The endometrial cancer tissue is typically depicted as hyper- or isoechoic relative to the surrounding myometrium (Fig. 1a) and cervical stroma invasion as thickened hyper- or isoechoic endometrium extending into the cervical canal and cervical stroma [33, 34, 35•]. The reported sensitivities (specificities) [accuracies] of TVU for the detection of deep myometrial invasion and cervical stroma invasion are 71–85 % (72–90 %) [72–84 %] and 29–93 % (92–94 %) [78–92 %], respectively (Table 2) [33, 34, 35•, 36, 37]. Due to the small field of view and limited depth of penetration using high-frequency vaginal ultrasound probes, TVU is not considered suited for valid assessment of pelvic and paraaortic lymph node metastases.

### Computed Tomography

Contrast-enhanced (CE) CT of the thorax, abdomen, and pelvis is widely employed preoperatively for the detection of lymph node metastases and distant spread in endometrial cancer. The primary tumors, when visible at CE CT, are typically depicted as slightly hypodense relative to the surrounding contrast-enhancing myometrial tissue (Fig. 1b). For local staging, CE CT has long been considered inferior to MRI and TVU [6] due to lower soft-tissue contrast resolution at CT, and recent literature reporting diagnostic performance for local staging parameters of CE CT is thus scarce (Table 2). A recent study of 24 endometrial cancer patients comparing CE CT with PET/CE CT for detection of pelvic and paraaortic lymph node metastasis also showed significantly lower sensitivity of CE CT than that of PET/CE CT, with reported sensitivities for lymph node metastases of 29 and 57 %, respectively [39].

### Magnetic Resonance Imaging

Pelvic MRI has long been established as a valuable imaging method in the preoperative staging of endometrial cancer [6, 44, 45]. Acquiring at least two T2-weighed sequences angled perpendicularly of the uterus is routinely performed; T1-weighted series with intravenous contrast is also normally included in the protocol due to the reportedly better diagnostic performance of CE MRI for identification of deep myometrial invasion than that of non-contrast MRI [6, 45]. The optimal contrast timing for diagnosing deep myometrial invasion is ∼2 min post contrast allowing the best discrimination between tumor tissue and the outer myometrial muscular layer [46].

Endometrial cancers are typically slightly hyperintense on T2-weighted images (Fig. 1c) and hypointense relative to the normal highly vascularized myometrium on CE T1 weighted images (Fig. 1d). The reported sensitivities (specificities) [accuracies] of CE MRI for the detection of deep myometrial invasion, cervical stroma invasion, and metastatic lymph nodes are 33–100 % (44–100 %) [58–100 %], 33–69 % (82–96 %) [46–89 %], and 17–80 % (88–100 %) [83–93 %], respectively, based on articles published in the last decade [37, 40, 41]. The broad range for these numbers on diagnostic performance illustrates that although MRI is considered one of the best imaging methods for preoperative staging in endometrial cancer, the diagnostic performance of CE MRI is still somewhat variable.

### FDG PET-CT

Fluorodeoxyglucose (18F-FDG) PET-CT combines two imaging techniques visualizing both morphologic and metabolic tumor characteristics at the same time-point allowing co-registration of structural and functional data depicted in fused images (Fig. 1g, h). PET-CT is increasingly employed in the preoperative staging of many cancers, including gynecologic cancers [7•, 37, 43, 47, 48]. The most common radiotracer is FDG, a glucose analogue that preferentially accumulates in malignant tissue due to its higher rate of glycolysis. Due to limitations of spatial resolution, FDG PET-CT is, however, unlikely to replace TVU and MRI for assessing pelvic disease state for depth of myometrial and cervical stromal invasion.

For the detection of lymph node metastases and distant spread, FDG PET-CT reportedly outperforms TVU and MRI, thus representing a very promising preoperative imaging method to differentiate between patients that are unlikely to benefit from lymphadenectomy and those that may profit from the procedure [47, 49]. The reported sensitivities (specificities) [accuracies] of FDG PET-CT in the detection of lymph node metastases are 74–85 % (91–96 %) [89–93 %] (Table 2) [7•, 37, 43]. The ability to correctly identify metastatic lymph nodes is, however, largely affected by lymph node size; node-based sensitivities of 100, 67, and 13 % in metastatic nodes ≥10, 5–9, and ≤4 mm, respectively, were reported in uterine cancers [50].

## Novel Imaging Methods for Preoperative Staging of Endometrial Cancer

Current intensive research efforts on novel imaging techniques as well as new contrast agents or tracers may provide improved imaging methods potentially enabling more accurate depiction of tumor extent and better detection of metastatic disease in endometrial cancer.

### 3D US

Three-dimensional (3D) ultrasound technology allows acquisition of ultrasound images from an organ or tissue of interest that may be reconstructed in any desired plane. This allows further analysis including virtual navigation and 3D ultrasound volume calculations of e.g., tumor volume [51]. The reported diagnostic performance of 3D ultrasound for assessment of deep myometrial invasion was quite promising in a study by Alcazar on endometrial cancer (*n* = 96) reporting a sensitivity (specificity) [accuracy] of 93 % (82 %) [85 %] [38]. Interestingly, applying a cut-off for the shortest tumor-free distance to serosa of ≤9 mm yielded a corresponding sensitivity (specificity) [accuracy] of 100 % (61 %) [72 %] for prediction of deep myometrial invasion [38].

### DW MRI

Diffusion weighted (DW) MRI is a functional imaging technique whereby contrast is derived from the random motion of water molecules [40]. Restricted diffusion of water molecules in the tissue is depicted as hyperintense on the high *b* value DW images (Fig. 1e) with corresponding hypointensity on the apparent diffusion coefficient (ADC) map (Fig. 1f). The diffusion property of the tumors is considered a surrogate marker for tumor cellularity as intact cells constitute a barrier to water diffusion [52]. Endometrial cancers typically exhibit restricted diffusion (Fig. 1e, f) with reported mean tumor ADC values in the range of 0.75–0.97 × 10^−3^ mm^2^/s, being significantly lower than that reported for benign uterine lesions (1.21–1.76 × 10^−3^ mm^2^/s) and normal endometrium/myometrium (1.52–1.71 × 10^−3^ mm^2^/s) [40]. The reported diagnostic performance of DW MRI for preoperative staging parameters in endometrial cancer is in the range of those reported for CE MRI (Table 1) [8, 40], and it has been advocated that CE MRI may be safely omitted when including DW MRI in patients in whom MRI contrast agents are contraindicated [53].

### MRI with Lymph Node-Specific Contrast Agent

The use of lymph node-specific contrast agent based on ultrasmall particles of iron oxide (USPIO) has been shown to dramatically improve the diagnostic performance of MRI for the detection of metastatic lymph nodes in endometrial and cervical cancer with reported sensitivity (specificity) [accuracy] of 91–100 % (87–94 %) [88–95 %] [42]. However, this contrast agent has unfortunately been withdrawn by the manufacturer pending further validation before potential implementation in the clinic.

## Potential Imaging Biomarkers in Endometrial Cancer

A biomarker is defined as a “characteristic that is objectively measured and evaluated as an indicator of normal biologic processes, pathogenic processes, or pharmacologic responses to a therapeutic intervention” [54]. Conventional imaging and novel functional imaging methods may be employed to visualize and quantify tumor extent as well as microstructural and functional tumor characteristics that are closely linked to clinical phenotype and histomorphological biomarkers. These imaging findings may serve as imaging biomarkers that may potentially aid to improve risk stratification and to tailor therapeutic strategy in endometrial cancer (Table 3).

**Table 3:** Potential preoperative imaging biomarkers in endometrial cancer

Imaging modality and/or parameter	Imaging characteristics of primary tumor predicting DMI and/or LNM and/or aggressive disease	Possible link between imaging biomarker and tumor pathophysiology	Proposed tumor cut-offs for risk stratification
TVU			
Echogenicity	Mixed or hypoechoic tumor predict DMI [11] Non-regular endometrial–myometrial borders [35•]	Tumor heterogeneity and altered tumoral texture	NR
Doppler parameters	High color score [11], low resistive index, and high peak systolic velocity [12], high VI [55]	Disorganized angiogenesis with altered tumoral blood flow	VI >7 for DMI and VI >10 for grade 3 tumors [55]
MRI			
ADC value (based on DW MRI)	Low ADCmean predicts DMI [8], high ADCq^a^ predicts DMI and LNM [13], and low ADCmin predicts aggressive disease [14]	Increased cellularity and intratumor heterogeneity of water movement	ADCmean < 0.75 for DMI [8] ADCmin < 0.66 for recurrence [14]
Blood flow (based on DCE-MRI)	Low tumor blood flow predicts reduced recurrence/progression-free survival [9•, 10]	Tumor hypoxia due to disorganized angiogenesis [9•]	NR
FDG PET-CT			
Metabolic parameters: SUVmax, SUVmean, MTV, and TLG	High tumor SUVmax, SUVmean, MTV, and TLG predict DMI, LNM, and poor prognosis [7•, 15•, 16, 17, 56, 57•, 58, 59]	Increasing metabolic activity of malignant tumors	MTV > 20 for DMI and MTV > 30 for LNM [7•] SUVmax > 9 [56] and >18 [17] and MTV > 9 and TLG > 70 [16] for high-risk MTV > 17 and TLG > 56 for recurrence [57•]
Tumor size (all imaging modalities)	Large tumor diameters and large tumor volume [7•, 16, 35•, 55, 57•, 60•, 61, 62]	Increased metastatic potential of large tumors	Volume index^b^ > 36 for LNM [62] and poor prognosis [61] AP diameter >2 cm for DMI and CC diameter >4 cm for LNM [60•] TFD ≤9 mm for DMI [38]

*ADC* apparent diffusion coefficient (10^−3^ mm^2^/s), *AP* anterioposterior, *CC* craniocaudal, *DCE* dynamic contrast enhanced, *DMI* deep myometrial invasion, *DW* diffusion weighted, *FDG* fluorodeoxyglucose, *LNM* lymph node metastases, *MRI* magnetic resonance imaging, *MTV* metabolic tumor volume (mL), *NR* not reported, *PET* positron emission tomography, *SUV* standard uptake value, *TFD* tumor-free distance to serosa, *TLG* total lesion glycolysis (g), *TV* transverse, *TVU* transvaginal ultrasound, *VI* vascularization index (%)

^a^ADCq is defined as the difference in ADC between the 25th and the 75th percentile voxel in one lesion [13]

^b^Volume index is defined as products of maximum anterioposterior (*AP*), transverse (*TV*), and craniocaudal (*CC*) diameters (cm) [62]

### Tumor Size

Pretherapeutic conventional imaging may yield clinically relevant information regarding primary tumor size (Table 2) [35•, 55, 60•, 61, 62]. The unfavorable prognostic impact of large tumor size in endometrial cancers is consistently supported by both in vivo and ex vivo studies [61–65], but optimal cut-off values for tumor size, based on preoperative imaging, for identification of high-risk patients are yet to be defined. A recent study of preoperative MRI in endometrial cancer patients found that anterioposterior (AP) tumor diameter >2 cm and craniocaudal (CC) tumor diameter >4 cm significantly predicted deep myometrial invasion and lymph node metastases, respectively; and that both tumor size parameters were significantly associated with reduced recurrence and progression-free survival [60•]. Similarly, volume index (defined as products of maximum AP, transverse (TV), and CC tumor diameters) >36 at preoperative MRI was reportedly associated with lymph node metastases [62] and dismal prognosis [61] in endometrial cancer. In line with this, tumor-free distance to serosa (TFD) ≤9 mm at TVU predicts deep myometrial invasion in endometrial cancer [38].

### Transvaginal Ultrasound Tumor Echogenicity and Doppler Parameters

Tumor echogenicity at preoperative TVU may provide additional information relevant for stage and prognosis in endometrial cancer. Mixed or hypoechoic tumors are reportedly more frequent in patients with deep myometrial invasion and in grade 3 tumors [11], and non-regular endometrial–myometrial border at TVU also predicts deep myometrial invasion [11, 35•]. Doppler parameters characterizing the vascular tumor morphology may also be linked to stage and grade. High color score and vascularization index (VI) are reportedly more frequent in tumors with deep myometrial invasion and in grade 3 tumors [11, 55]. One study proposed cut-offs for the vascularization index for the prediction of deep myometrial invasion and grade 3 tumors of VI >7 and VI >10 %, respectively [55].

### ADC Measurements Reflecting Tumor Microstructure

DW MRI with measurements of tumor ADC values may provide additional information about tumor microstructure with potential relevance for staging and prediction of aggressive disease. Low mean tumor ADC value is associated with deep myometrial invasion [8]. Interestingly, high ADCq (defined as the difference in ADC between the 25th and the 75th percentile voxel in one lesion), putatively reflecting high intratumor heterogeneity of water movement, is associated with deep myometrial invasion, cervical involvement, lymph node metastases, and lymphovascular space invasion [13]. Similarly, minimum ADC value (ADCmin) of the primary tumor is reportedly lower in patients with deep myometrial invasion, cervical involvement, and lymph node metastases, and in patients with grade 3 endometrioid subtype [14]. Furthermore, ADCmin significantly predicted reduced disease-free survival, also after adjusting for FIGO stage [14], suggesting that tumor ADC measurements may potentially yield additional prognostic information aiding in risk stratification when selecting patients for adjuvant treatment.

### DCE-MRI Parameters Reflecting Tumor Microvasculature

Dynamic contrast-enhanced (DCE) MRI is a novel functional imaging technique allowing quantitative assessment of tissue perfusion and vascular permeability, enabling characterization of tumor microvasculature and the angiogenic profile of tumor tissue in vivo [66]. Recent findings suggest that DCE-MRI tumor parameters are significantly linked to specific clinical and histological phenotypes in endometrial cancer [9•, 10]. Low tumor Fb (blood flow) and high tumor E (extraction fraction; reflecting capillary leakage) predict reduced recurrence/progression-free survival and are more frequent in non-endometrioid tumors [10]. Interestingly, Fb is also reportedly inversely correlated to the expression of prognostic immunohistochemical markers reflecting microvascular proliferation [9•]. Tumor hypoxia, which is a characteristic feature of various solid tumors and believed to promote tumor progression and resistance to therapy [67, 68], may thus play a pivotal role in the pathogenic mechanisms leading to tumor growth and metastatic spread, in endometrial cancer.

### FDG PET-CT Parameters Reflecting Tumor Metabolism

Paralleling the well-documented feasibility of FDG PET-CT for detection of lymph node metastases in endometrial cancer, the potential value of FDG PET-specific quantitative tumor parameters for predicting clinical and histologic tumor characteristics in endometrial cancer has been increasingly explored [7•, 15•, 16, 17, 56, 57•, 58, 59, 69]. Tumor maximum standard uptake value (SUVmax) is the most frequently reported PET parameter; SUVmax representing the value of the voxel with the highest SUV within the drawn volume of interest (VOI) putatively represents tumor tissue (Fig. 1g, h) [7•]. The VOI is typically manually drawn using prespecified thresholds for SUV (e.g., SUV > 2.5) of voxels to be included in the VOI, and metabolic tumor volume (MTV) and mean SUV (SUVmean) are calculated in this VOI. Total lesion glycolysis (TLG), which is derived from SUVmean and MTV (TLG = SUVmean × MTV), represents a measure of total viable tumor cells within the tumor, and TLG is increasingly reported in studies on endometrial cancer [7•, 16, 57•, 70].

High-tumor SUVmax, SUVmean, MTV, and TLG are uniformly reported to predict deep myometrial invasion, cervical stroma invasion, lymph node metastases, and poor prognosis in endometrial cancer [7•, 15•, 16, 17, 56, 57•, 58, 59, 69]. The proposed cut-offs for these parameters to identify high-risk patients have, however, a relatively wide range in the literature: for SUVmax >9–18 [17, 56], for MTV >9–30 mL [7•, 16, 57•], and for TLG >56–70 g [16, 57•] (Table 3). This variation in proposed cut-offs for predicting high-risk phenotype may be due to dissimilar patient cohorts and lack of standardization of imaging protocols and post-processing methods (e.g., manual ROI placement and different threshold for SUV to be included in the MTV) in the studies. Thus, further studies are needed to validate and better standardize metabolic imaging parameters including optimized thresholds for risk stratification for potential clinical use.

### In Vivo MR Spectroscopy

In vivo MR spectroscopy (MRS) is a method to obtain biochemical information non-invasively from biological tissue. Within a selected volume of interest, typically tumor tissue, signals from chemical nuclei in the tissue are registered; the most commonly used nuclei are protons (hydrogen). MRS has long been established as a valuable adjunct to conventional MRI in the assessment of various tumors, e.g., tumors in the brain, prostate, and breast [71]. Studies on MRS in endometrial cancer are scarce, but some studies have reported increased signals from choline in endometrial cancer tumors [72–75]. Interestingly, a recent study found that the choline/water ratio increased with increasing tumor stage and large tumor size in endometrial cancer [74]. Furthermore, another choline-derived parameter, choline signal to noise ratio (ChoSNR), is reportedly significantly higher in type 2 endometrial cancers than that in type 1 endometrial cancer [75]. Altered choline profile in endometrial cancer has also been demonstrated using high-resolution magic angle spinning (HR-MAS) ^1^H nuclear magnetic resonance (NMR) techniques on endometrial cancer biopsies [76], confirming the central role of choline in the metabolic rearrangements subsequent to malignant transformation in endometrial cancer. The potential value of choline as biomarker based on in vivo MRS or HR-MAS of biopsies in endometrial cancer is, however, largely unknown.

### Textural Imaging Features

Texture analysis is an image post-processing technique analyzing a set of quantified metrics to assess the spatial arrangements of densities/intensities in a volume of interest. Quantitative measures of image heterogeneity have been shown to be closely linked to tissue markers of heterogeneity, hypoxia, and angiogenesis and have also been shown to predict survival for various cancers [77]. Whether texture analysis of VUS, CT, MRI, and PET may provide imaging biomarkers in endometrial cancer is not yet established.

### Novel PET Tracers

A wide range of novel PET radiotracers is currently being developed with the aim of imaging relevant biological processes and molecular targets in clinical oncology [78]. However, there is currently very limited experience with the use of these novel tracers in endometrial cancer. PET imaging of endometrial cancer with tracers specific for e.g., hypoxia, cell proliferation, amino acid metabolism, angiogenesis, apoptosis, blood flow, fatty acid metabolism, or estrogen receptors may, however, lead to increased understanding of the biologic processes relevant for tumor progression and metastatic spread in endometrial cancer, and will be particularly interesting to explore as predictive markers sequentially during treatment with targeted and novel therapeutics for early signs of response. Still, the implementation of novel PET tracers in endometrial cancer is largely awaited.

## Interobserver Agreement for Preoperative Staging and Reproducibility of Imaging Biomarkers

High interobserver agreement is crucial for evaluating the usefulness of a diagnostic test, and the interobserver agreement should ideally be systematically evaluated before introduction of the test in the clinic. Quite variable interobserver agreement for the evaluation of deep myometrial invasion by VUS has been observed both among ultrasound experts and among general gynecologists with reported kappa values of 0.24–0.81 and 0.26–0.71, respectively [79]. For VUS assessment of cervical stroma invasion, the interobserver agreement was also variable, however, with significantly better agreement reported for ultrasound experts (kappa values of 0.35–0.77) than for general gynecologists (kappa values of 0.05–0.75) [79]. Varying interobserver agreement is also observed at MRI for the evaluation of deep myometrial invasion, cervical stroma invasion, and lymph node metastases with reported kappa values of 0.16–0.91, 0.46–0.77, and 0.36–0.74, respectively [8, 41, 80, 81].

The reproducibility of tumor measurements that may be used as potential imaging biomarkers should be thoroughly assessed prior to implementation in the clinic, and the interobserver agreement for these measurements should ideally be very good to warrant inclusion in risk stratification models. VUS measurements of tumor-free distance (TFD) to serosa in endometrial cancer reportedly yield very good interobserver agreement with ICC of 0.91 [38]. For tumor size measurements at MRI, the reported interobserver agreement is also very good with ICC of 0.78–0.85 [60•], and tumor ADC value measurements seem also quite robust with an ICC of 0.60 [8]. Reported ICC for measurements of SUVmax, SUVmean, MTV, and TLG in endometrial cancer is 0.98, 0.87, 0.56, and 0.57, respectively [7•]. The moderate agreement observed for MTV and TLG measurements may be due to the subjective steps involved in the manual placement of the VOI for estimation of MTV and TLG.

## Conclusions

Preoperative imaging is crucial in order to enable tailored surgical procedure in endometrial cancer. Whereas TVU and/or pelvic MRI are preferred for the assessment of local pelvic tumor extent, PET-CT and/or CT may improve the detection of lymph node metastases and distant spread. All imaging methods are, however, hampered by non-perfect accuracies for the staging parameters and limitations in reproducibility. Novel structural and functional imaging techniques, visualizing microstructural and functional tumor characteristics, may be closely linked to clinical phenotype, tumor stage, and tumor biologic characteristics in endometrial cancer. Such characteristics based on novel imaging techniques may thus serve as future imaging biomarkers in endometrial cancer. Importantly, potential new imaging biomarkers should be thoroughly assessed for reproducibility and studied in relation to currently standardly applied preoperative biomarkers from e.g., endometrial biopsies, also documented to be hampered by non-perfect accuracies in predicting disease spread and poor outcome, and with well-documented limitations in reproducibility. The potential added value from novel imaging technique will need to be explored in the context of the currently applied state of the art methods for preoperative risk assessment to tailor endometrial carcinoma treatment, also assessing costs and benefits for different approaches to elucidate potential clinical benefit from advanced imaging methods implemented in clinical care.
